# The Transcription Factor SsZNC1 Mediates Virulence, Sclerotial Development, and Osmotic Stress Response in *Sclerotinia sclerotiorum*

**DOI:** 10.3390/jof10020135

**Published:** 2024-02-08

**Authors:** Yongkun Huang, Zhima Zhaxi, Yanping Fu, Jiatao Xie, Tao Chen, Bo Li, Xiao Yu, Yang Lin, Daohong Jiang, Jiasen Cheng

**Affiliations:** 1 National Key Laboratory of Agricultural Microbiology, Huazhong Agricultural University, Wuhan 430070, China; ykn.wong@webmail.hzau.edu.cn (Y.H.);; 2 The Provincial Key Laboratory of Plant Pathology of Hubei Province, College of Plant Science and Technology, Huazhong Agricultural University, Wuhan 430070, China; 3 Hubei Hongshan Laboratory, Wuhan 430070, China

**Keywords:** *Sclerotinia sclerotiorum*, Zn_2_Cys_6_ proteins, SsZNC1, pathogenesis, sclerotial development, abiotic stresses

## Abstract

*Sclerotinia sclerotiorum* is a fungal pathogen with a broad range of hosts, which can cause diseases and pose a great threat to many crops. Fungal-specific Zn_2_Cys_6_ transcription factors (TFs) constitute a large family prevalent among plant pathogens. However, the function of Zn_2_Cys_6_ TFs remains largely unknown. In this study, we identified and characterized SsZNC1, a Zn_2_Cys_6_ TF in *S. sclerotiorum*, which is involved in virulence, sclerotial development, and osmotic stress response. The expression of *SsZNC1* was significantly up-regulated in the early stages of *S. sclerotiorum* infection on *Arabidopsis* leaves. The target deletion of *SsZNC1* resulted in reduced virulence on *Arabidopsis* and oilseed rape. In addition, sclerotial development ability and growth ability under hyperosmotic conditions of *SsZNC1* knockout transformants were reduced. A transcriptomic analysis unveiled its regulatory role in key cellular functions, including cellulose catabolic process, methyltransferase activity, and virulence, etc. Together, our results indicated that SsZNC1, a core regulatory gene involved in virulence, sclerotial development and stress response, provides new insight into the transcription regulation and pathogenesis of *S. sclerotiorum*.

## 1. Introduction

*Sclerotinia sclerotiorum* is a fungal pathogen that is widely infective in crops such as oilseed rape, soybean, and sunflower, causing yield and quality losses, thereby seriously affecting agricultural productivity [1,2]. The annual cost of *S. sclerotiorum* in the United States exceeds USD 200 million [3,4]. *S. sclerotiorum* infects sunflowers, causing USD 100 million in yield losses and diminished quality [5]. *S. sclerotiorum* has a distinctive infection strategy characterized by the production of sclerotia. Sclerotia serve as enduring reservoirs for the pathogen, enabling its persistence in the soil and facilitating subsequent infection cycles [6]. Under suitable temperature and humidity conditions, sclerotia can germinate, produce ascospores that are released to initiate host infection, or directly germinate into hyphae to infect plants [6]. The pathogenesis of *S. sclerotiorum* is relatively complex. Research mainly focuses on its secreted hydrolases, oxalic acid, early secretory pathways, and secreted proteins [7,8,9,10]. However, the biological function and mechanism of transcription factors (TFs) in the pathogenic process of *S. sclerotiorum* remain relatively understudied.

TFs are a class of proteins that regulate gene expression by recognizing a specific DNA sequence within regulatory regions, thus modulating the initiation of transcription by RNA polymerase [11]. TFs play crucial regulatory roles in various biological processes in fungi, including growth, development, abiotic stress response, pathogenicity, and environmental adaptation [12,13]. Typically, fungal TFs contain specific domains such as DNA-binding domains and activation regulatory domains, enabling them to interact with specific DNA sequences [12]. Through these interactions, fungal TFs modulate the expression levels of downstream genes. According to different DNA-binding domains, TFs are divided into different families, including zinc finger, homeodomain or homeobox (HD/Hox), basic leucine-zipper (bZIP), and basic helix–loop–helix (bHLH), etc. [14]. The zinc finger family is one of the largest classes of TFs in fungi and mainly includes Cys_2_His_2_ (C_2_H_2_), Cys_4_ (GATA), and Zn_2_Cys_6_ proteins [14,15]. Zn_2_Cys_6_ TFs have been studied in some plant pathogens [16,17,18,19], but there are relatively few studies in *S. sclerotiorum*. Gaining insight into the functions of Zn_2_Cys_6_ TFs contributes to a better comprehension of the pathogenesis of *S. sclerotiorum*.

Over the years, several studies have shed light on the pivotal role of TFs in the regulatory network of *S. sclerotiorum*. SsNsd1 is a GATA-type zinc-finger TF in *S. sclerotiorum*, crucial for regulating asexual–sexual development, appressoria formation, and virulence [20,21]. SsNsd1 interacts with a formaldehyde dehydrogenase, SsFdh1, influencing formaldehyde detoxification, nitrogen metabolism, and virulence [22]. A forkhead-box (FOX) TF, SsFoxE3, plays a crucial role in sclerotia, compound appressoria formation, and pathogenicity, positively regulating detoxification- and autophagy-related genes [23]. SsFKH1, an atypical FOX TF, is involved in sclerotial development and virulence [24]. SsFKH1 interacts with MAP kinase SsMkk1, potentially serving as its downstream substrate [25]. SsSte12, a key TF in the MAPK pathway of *S. sclerotiorum*, plays a crucial role in mycelium growth, sclerotial development, appressoria formation, and virulence [26,27]. SsZFH1, a C_2_H_2_ TF in *S. sclerotiorum*, is essential for sclerotial and apothecia development, influencing mycelium growth and oxidative stress response, with implications for lesion expansion on diverse host plants [28]. While some studies have been conducted to understand the functions of *S. sclerotiorum* TFs, there are still many TFs, especially Zn_2_Cys_6_ TFs, with undetermined functions that require further exploration and investigation.

In this study, we focused on the function of Zn_2_Cys_6_ TFs in *S. sclerotiorum*. Based on public transcriptome data [29], we found that *SsZNC1* exhibited a relatively high expression among Zn_2_Cys_6_ TFs during *S. sclerotiorum* infection, so this gene became our research target. SsZNC1 had a GAL-4-like Zn_2_Cys_6_ DNA-binding domain, and its homologous were widely distributed in ascomycetes. The expression of *SsZNC1* was up-regulated during infection, peaking in the early stages. The targeted deletion and complementation of *S. sclerotiorum SsZNC1* revealed its role in virulence, sclerotial development, and osmotic stress response. An RNA-seq analysis highlighted its regulatory impact on key biological processes. This study on the TF SsZNC1 not only enhances our understanding of its multifunctional roles in *S. sclerotiorum*, but also provides insights into potential targets for disease management.

## 2. Materials and Methods

### 2.1. Identification and Sequence Analysis of SsZNC1

Sequences of *SsZNC1* (*sscle_12g087860*) and its homologous were retrieved from the NCBI GenBank database. The conserved domains of SsZNC1 were predicted by NCBI Conserved Domain Search Tools [30]. The SsZNC1 promoter was predicted by using the Promoter 2.0 Prediction Server [31]. The alignment of SsZNC1 and its homologous protein sequences was conducted using ClustalW (version 2.0.10). The phylogenetic tree was constructed with MEGA 11 [32] using the maximum-likelihood method with 1000 replicates for a bootstrap analysis. The evolutionary history was inferred by using the Maximum Likelihood method and JTT matrix-based model. The percentage of trees in which the associated taxa were clustered together was analyzed and shown next to the branches. The *Verticillium dahliae* sequence (RXG48842.1) was used as the outgroup.

### 2.2. Fungal Strains and Plant Materials

The *Sclerotinia sclerotiorum* wild-type strain 1980 (ATCC 18683), *SsZNC1* knockout mutant (Δ*SsZNC1*), and complemented strain (Δ*SsZNC1*-C) were cultivated on potato-dextrose agar (PDA) plates at 20 °C in the dark. The *Arabidopsis thaliana* Col-0 wild-type seeds were surface sterilized with alcohol for 2 min, then with 5% sodium hypochlorite for 5 min, and washed three times with sterile water. The seeds were grown on a half-strength Murashige & Skoog plate at 4 °C for 3 days [33]. The plates were cultivated in a growth room at 20 °C with a 12 h light/12 h dark photoperiod for 10 days. Seedlings were transplanted into the soil in a growth room. Oilseed rape (*Brassica napus*) was grown in a growth chamber under a 16 h light/18 h dark photoperiod at 25 °C. Four-week-old *Arabidopsis* leaves and six-week-old oilseed rape leaves were used for pathogenicity assays.

### 2.3. SsZNC1 Gene Deletion and Complementation

The deletion of the SsZNC1 gene was accomplished through the implementation of the split marker system [34]. The deletion strategy for *SsZNC1* is elucidated in Figure S1A. The 5′ and 3′ flanking fragments of the *SsZNC1* ORF were amplified from the genomic DNA of the *S. sclerotiorum* strain 1980. Two truncated sequences of the hygromycin-resistant gene were amplified from the pUCH18 plasmid. Subsequently, the 5′ and 3′ flanking fragments of the *SsZNC1* ORF were individually ligated with the respective truncated resistance gene sequences. These two connected fragments were separately constructed on the pMD19-T plasmid. The two connected fragments were then amplified and utilized for protoplast transformation. Protoplast transformation was performed as described by Rollins [35]. *S. sclerotiorum* hyphae were subjected to cell wall lysis using 0.015 g/mL of lysing enzymes (L1412, Sigma, St. Louis, MO, USA) for 1 h at 30 °C, and then the protoplasts were harvested by filtration and centrifugation (3000× *g*, 10 min). The two fragments were introduced into the protoplasts via PEG-mediated protoplast transformation. The knockout mutants of the *SsZNC1* gene were selected on plates containing hygromycin and confirmed through PCR. Homozygous transformants were obtained through single ascospore isolation.

For *SsZNC1* complementation, the *SsZNC1*-*3* × *FLAG* with its native promoter region was amplified and constructed into a pCETNS plasmid. Sequences from promoter to terminator and the G418-resistance sequence were amplified from this vector. Fragments were purified and used for protoplast transformation. Transformants were selected on plates containing G418 and confirmed through PCR, RT-PCR, and Western blot analysis.

### 2.4. Phenotypic Analysis

To evaluate the growth rates of both the WT and transformants, activated strains were cultivated on PDA for 2 days. Then, the mycelium diameter was measured and the hyphae were observed under a microscope. For sclerotia production ability, WT and *SsZNC1* transformants were grown on PDA for 15 days and then the sclerotia morphology, sclerotia quantity, and quality were recorded. For an infection cushion assay, inoculated mycelium plugs of the WT and transformants on the Parafilm were grown for 2 days. The formation of the infection cushion was observed under a microscope and the number of infection cushions was counted. For determination of the acid production capacity, WT and *SsZNC1* transformants were grown on PDA medium containing 0.005% (*w*/*v*) bromophenol blue dye and photographs were taken at 48 h post inoculation (hpi).

To detect the growth of strains under stress, mycelium plugs (4 mm diameter) were cultured on PDA containing several separate stress factors, including 1.2 M sorbitol, 0.5 M NaCl, 0.02% SDS, and 2.5 mg/mL Congo red. To obtain suitable photos of the hyphae, photos of different factors were taken at 60 hpi, 60 hpi, 72 hpi, and 48 hpi, respectively. The inhibition rate of hyphal growth was calculated by measuring the hyphal diameter every 12 h. All the above experiments were repeated three times, with three biological replicates for each repeat.

### 2.5. Pathogenicity Assay

The *S. sclerotiorum* WT, *SsZNC1* gene knockout, and complementation strains were grown on PDA at 20 °C for 2 days. Hyphal plugs (2 mm or 5 mm diameter) from colony margins were placed on four-week-old *Arabidopsis* leaves or six-week-old oilseed rape leaves and the pathogenicity was assessed at 36 hpi or 48 hpi. Lesion sizes were quantified using ImageJ software (version 1.52a). The relative biomass was quantified by qPCR through DNA extraction from equal areas of individual infected leaves. The qPCR was performed using TransStart^®^ Green qPCR SuperMix (AQ101, TransGen Biotech, Beijing, China). The qPCR program was initiated at 94 °C for 30 s, followed by a cycling stage at 94 °C for 5 s and at 60 °C for 15 s, with a total of 42 cycles. The primers used are listed in Table S1.

### 2.6. Transcriptomic Analysis

The total RNA was extracted from the mycelial samples collected at 3 hpi and 9 hpi for both the *S. sclerotiorum* WT and the knockout mutant Δ*SsZNC1*. Each treatment group included three independent replicates to ensure the reliability of the experimental results. The RNA samples were subjected to high-throughput sequencing using the Illumina HiSeq instrument (AZENTA, Beijing, China). Raw sequencing data were processed to obtain clean reads by filtering out low-quality reads and adapter sequences. Clean reads were then aligned to the reference genome of *S. sclerotiorum* using the bioinformatics tool HISAT2 (version 2.2.1). The expression levels of the genes were quantified, and a differential expression analysis was performed to identify genes with significant changes in expression between the WT and *SsZNC1* knockout mutant at 3 hpi and 9 hpi. A fold change cutoff of ≥two-fold and a *p*-value of ≤0.05 were used to determine differentially expressed genes (DEGs) using the edgeR package (version 3.12.1) with TMM normalization. The DEGs were input into the Blast2GO (version 2.2.31) program for a Gene Ontology (GO) terms classification. The enrichment analysis of the DEGs in the GO terms and Kyoto Encyclopedia of Genes and Genomes (KEGG) pathways were conducted using the phyper, clusterProfiler, and pathview bioinformatics functions in the R software (version 4.3.2) [36,37].

### 2.7. RNA Extraction and Quantitative Real-Time PCR Analysis

The *S. sclerotiorum* WT and *SsZNC1* knockout mutant were inoculated onto four-week-old *A. thaliana* leaves. Mycelial samples were collected at 3 hpi and 9 hpi for RNA extraction. The total RNA from the samples was reverse transcribed into cDNA using a cDNA synthesis supermix kit (AT311-02, TransGen Biotech, Beijing, China) according to the manufacturer’s instructions. For the RT-qPCR analysis, gene-specific primers are listed in Table S1. The transcript levels were calculated by the 2^−ΔΔCt^ method. Each experiment was repeated three times.

### 2.8. Statistical Analysis

Statistical analyses were performed using the one-way ANOVA method with IBM SPSS Statistics 26 to evaluate the significance of differences. Differences at *p* < 0.01 or *p* < 0.05 were both considered as statistically significant. All experiments were repeated with at least three replicates.

## 3. Results

### 3.1. Identification and Analysis of SsZnNC1

**Figure 1:**
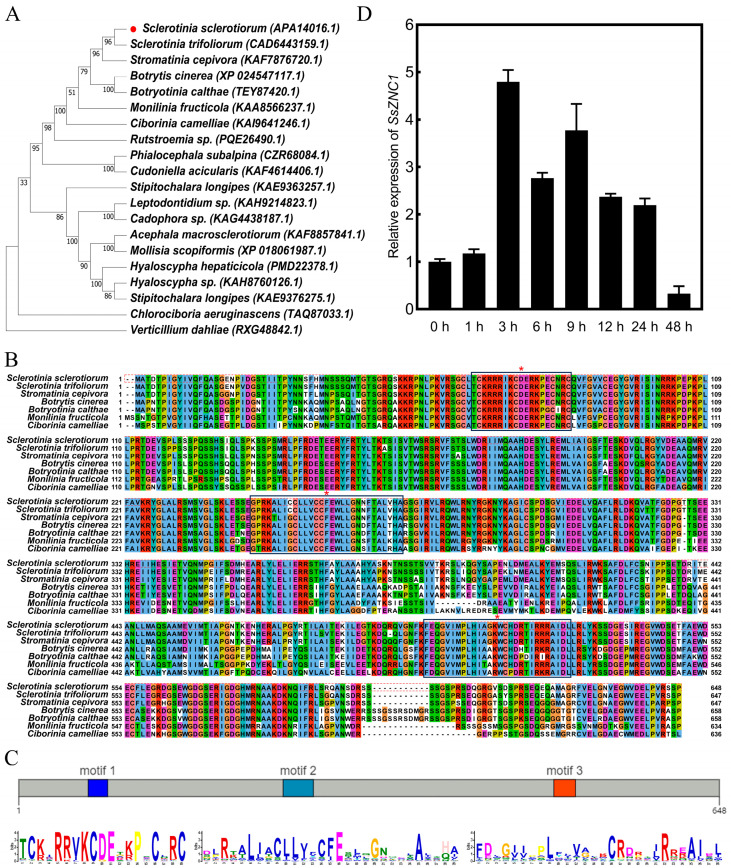
Phylogenetic analysis, sequence conserved alignment, and expression pattern of *SsZNC1*. (**A**) Phylogenetic analysis of SsZNC1. The phylogenetic tree was constructed using MEGA11 using the maximum likelihood method. *Sclerotinia sclerotiorum* sequence (APA14016.1) was marked with red circle and *Verticillium dahliae* sequence (RXG48842.1) was used as the outgroup. (**B**) Amino acid sequence alignment of SsZNC1 and its homologous from six different species using Jalview software (version 2.11.3.2). *S. sclerotiorum* sequence was shown in red dotted line box. Black solid line box of tag (*) indicated the three conserved motifs in the alignment. (**C**) Conserved motifs of SsZNC1 predicted by MEME suite (version 5.5.5). In total, 250 *SsZNC1* and its homologous amino acid sequences were used for analysis. (**D**) Relative levels of transcript accumulation of *SsZNC1* were assessed through RT-qPCR in inoculated *Arabidopsis thaliana* leaves for 0–48 h. Values are the means of three independent trials.

To investigate the biological function of the Zn_2_Cys_6_ transcription factor (TF) in *S. sclerotiorum*, we selected *SsZNC1* for study based on the published transcriptome data [29]. *SsZNC1* had a full-length sequence of 2008 bp with two exons, encoding a protein of 648 amino acids, and the predicted protein size was 73.0 kDa. The SsZNC1 protein contained a GAL4-like Zn_2_Cys_6_ binuclear cluster DNA-binding domain, suggesting that SsZNC1 may function as a TF. SsZNC1 orthologues were widely distributed among ascomycetes (Figure 1A). A sequence alignment analysis showed that the SsZNC1 protein sequence had a high sequence similarity with the homologous of *Sclerotinia trifoliorum*, *Stromatinia cepivora*, *Botrytis cinerea*, *Botryotinia calthae*, *Monilinia fructicola* and *Ciborinia camlliae* (Figure 1B). Their identity percentages were 95%, 92%, 77%, 76%, 73%, and 70%, respectively. SsZNC1 contained three conserved motifs, and the peptide TCKXRRIKCDE in motif 1 was highly conserved in 250 homologous of SsZNC1 (Figure 1C). To understand the function of SsZNC1 during *S. sclerotiorum* infection, the expression pattern of *SsZNC1* was detected by RT-qPCR. The results showed that the expression level of SsZNC1 increased after inoculation, peaked at 3 h post-inoculation (hpi), and gradually decreased after 9 hpi (Figure 1D).

### 3.2. Targeted Deletion and Complementation of SsZNC1 in S. sclerotiorum

To explore the biological function of the TF SsZNC1, we used homologous recombination technology and the split-marker method to knock out the gene (Figure S1A). Homozygous knockout mutants of *SsZNC1* were obtained by ascospore isolation. To obtain the complemented strain, homozygous knockout transformants were complemented by inserting the complete gene fragment with its own promoter and 3 × FLAG tag into the genome. Two *SsZNC1* knockout mutants (Δ*SsZNC1*-1 and Δ*SsZNC1*-2) and two complemented strains (Δ*SsZNC1*-1C-1 and Δ*SsZNC1*-1C-2) were obtained. The PCR analysis results showed that the *SsZNC1* gene sequence could not be amplified in the two knockout transformants, but it was amplified in both the WT and complemented strains (Figure S1B). Both knockout mutants and complemented strains could amplify the flanking sequences of *SsZNC1* (Figure S1B). To verify the changes in the transcription level of the transformants, we used RT-PCR analysis, and the results showed that the sequence of *SsZNC1* could not be detected in the knockout mutants, while the target band could be verified in both the WT and complemented strains (Figure S1C). To validate the reinstatement of the SsZNC1 protein expression in the complemented strain, a Western blot analysis was conducted. The results showed that the protein band was twice as large as expected (Figure S1D). The above results demonstrate the complete deletion of the gene sequence in the knockout transformants and comprehensive complementation of the complemented strain.

### 3.3. SsZNC1 Is Important for Sclerotial Development and Coping with Hyperosmotic Stress

**Figure 2:**
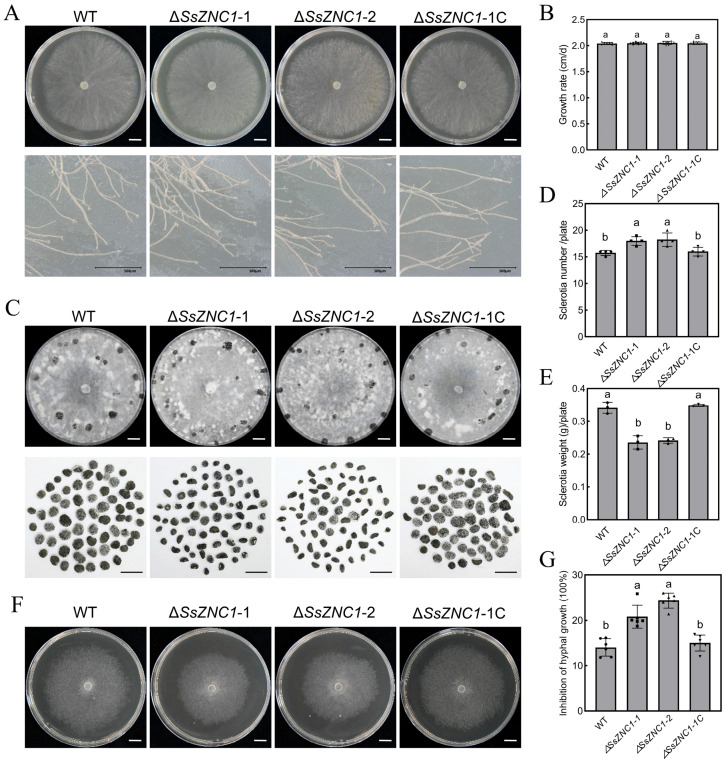
The deletion of *SsZNC1* has significant effects on sclerotial development and hyperosmotic stress tolerance but no effects on hyphae morphology. (**A**) Colony morphology and hyphal tips of the wild-type (WT) and the *SsZNC1* transformants grown on PDA at 20 °C. Photos were taken at 48 hpi. Upper graph bar, 1 cm. Lower graph bar, 500 μm. (**B**) The growth rate of the WT and the *SsZNC1* transformants on PDA for 48 h at 20 °C. (**C**) The sclerotial morphology of the WT and the *SsZNC1* transformants on PDA for 15 d at 20 °C. Sclerotia were collected from four plates (9 cm diameter). Bar, 1 cm. (**D**) The sclerotial number of the WT and the *SsZNC1* transformants. (**E**) The sclerotial dry weight of the WT and the *SsZNC1* transformants. (**F**) Growth observation of the WT and the *SsZNC1* transformants on PDA medium containing 1.2 M sorbitol. Bar, 1 cm. (**G**) The inhibition of hyphal growth of the WT and the *SsZNC1* transformants on PDA medium containing sorbitol. The values represent means derived from three independent replicates, and the error bars indicate ±SD of the mean. Different lowercase letter (a,b) represented significant differences between groups (*p* < 0.01).

To determine the function of SsZNC1, we examined the biological phenotypes of the knockout and complemented strains. Compared with the WT, two *SsZNC1* knockout mutants (Δ*SsZNC1*-1 and Δ*SsZNC1*-2) showed no significant differences in their colony and hyphal tip morphology on the PDA plate (Figure 2A). There were also no significant differences in growth rate (Figure 2B). However, knockout mutants produced smaller and more sclerotia than the WT and complemented strains after 15 d on PDA (Figure 2C). The number of sclerotia generated by the knockout mutants increased by approximately 15%, but the weight decreased by about 30% (Figure 2D,E).

Tolerance to environmental stresses is very important for pathogens to infect hosts. To explore whether SsZNC1 is involved in the response of *S. sclerotiorum* to environmental stresses, we inoculated the WT, *SsZNC1* knockout mutants, and complemented strain on PDA plates supplemented with different stress. Two knockout mutants grew significantly slower than the WT and complemented strains on PDA containing high concentrations of sorbitol (Figure 2F). The average inhibition rate of the two knockout mutants was 23%, compared with 14% for the WT and complemented strains (Figure 2G). However, the knockout mutants showed no significant differences in hyphal morphology and growth inhibition rates regarding their tolerance to NaCl, SDS, and Congo red compared to the WT and complemented strains (Figure S2). The above results indicate that SsZNC1 is involved in sclerotial development and hyperosmotic stress response.

### 3.4. SsZNC1 Plays an Important Role in Virulence

**Figure 3:**
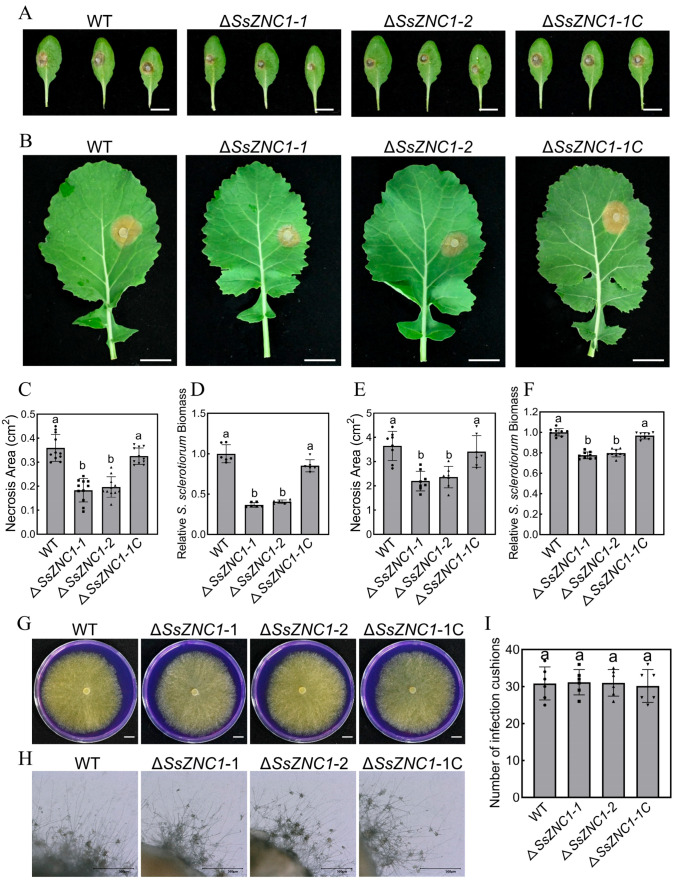
*SsZNC1* is important for full virulence of *S. sclerotiorum* but not for oxalate production and infection cushion formation. (**A**) Pathogenicity phenotype of the WT and *SsZNC1* transformants on *A. thaliana* leaves. Pictures were taken by 36 hpi. Bar, 1 cm. (**B**) Pathogenicity phenotype of the WT and the *SsZNC1* transformants on oilseed rape leaves. Pictures were taken by 48 hpi. Bar, 2 cm. (**C**) Necrosis area on *A. thaliana* leaves. (**D**) Relative *S. sclerotiorum* biomass on *A. thaliana* leaves. (**E**) Necrosis area on oilseed rape leaves. (**F**) Relative *S. sclerotiorum* biomass on oilseed rape leaves. (**G**) Qualitative determination of acid produced by the WT and the *SsZNC1* transformants on PDA containing 0.005% (*w*/*v*) bromophenol blue dye at 20 °C. Photographs were taken at 48 hpi. Bar, 1 cm. (**H**) The infection cushion phenotypes of the WT and the *SsZNC1* transformants on Parafilm at 48 hpi. Bar, 500 μm. (**I**) The infection cushion numbers of the WT and the *SsZNC1* transformants. The values represent means derived from three independent replicates, and the error bars indicate ± SD of the mean. Different lowercase letter (a,b) represented significant differences between groups (*p* < 0.01).

To investigate the biological function of SsZNC1 in the pathogenesis of *S. sclerotiorum*, we evaluated the virulence of the Δ*SsZNC1* mutants on the leaves of *Arabidopsis thaliana* and *Brassica napus*. Compared with the WT strain, the *SsZNC1* knockout mutants caused smaller lesions on the *Arabidopsis* leaves and oilseed rape (Figure 3A,B). The complemented strain restored virulence to the level of the WT strain (Figure 3A,B). The lesion areas produced by two knockout mutants in the *Arabidopsis* leaves were approximately 0.18 cm^2^ and 0.19 cm^2^, while the lesion area produced by the WT strain was about 0.36 cm^2^ (Figure 3C). The relative *S. sclerotiorum* biomass of the knockout mutants was reduced by 61.4% on average (Figure 3D). When inoculated on oilseed rape leaves at 48 hpi, the lesion areas and relative *S. sclerotiorum* biomass of the knockout mutants decreased by approximately 37.5% and 21.4%, respectively (Figure 3E,F). To explore the role of SsZNC1 in regulating *S. sclerotiorum* virulence, we tested whether SsZNC1 affected acid production and infection cushion formation. The knockout mutants showed no significant differences from the WT and complemented strains in acid production on PDA containing bromophenol blue and infection cushion formation (Figure 3G–I). These results indicate that SsZNC1 is involved in the pathogenesis of *S. sclerotiorum*, and its involvement is not attributed to influencing acid production and infection cushion formation.

### 3.5. RNA-Seq Analysis of the ΔSsZNC1 Mutant

**Figure 4:**
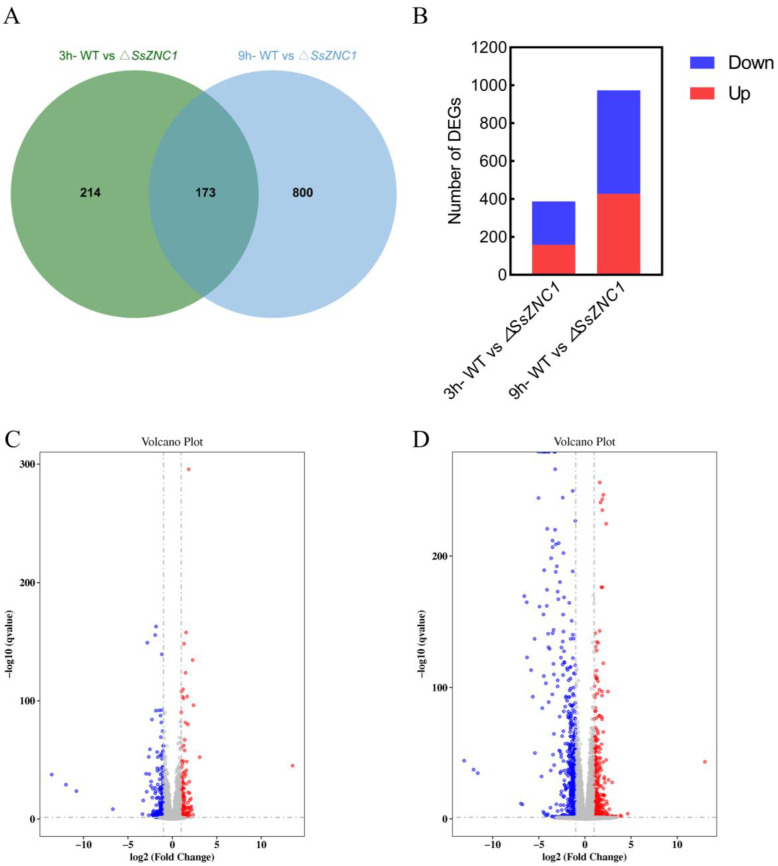
The number of differential expression genes (DEGs) in different comparable groups. (**A**) The Venn diagram of *S. sclerotiorum* DEGs when WT vs. Δ*SsZNC1* infected *Arabidopsis* leaves at 3 hpi and 9 hpi. (**B**) The number of up- and down-regulated DEGs in WT vs. Δ*SsZNC1* at 3 hpi and 9 hpi. (**C**) Volcano maps show the distribution of DEGs for WT vs. Δ*SsZNC1* at 3 hpi. (**D**) Volcano maps show the distribution of DEGs at 9 hpi.

**Figure 5:**
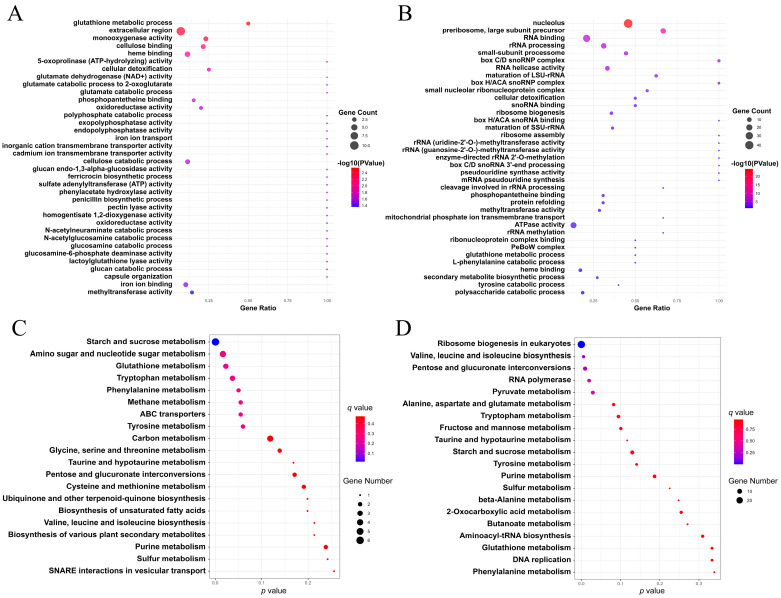
Analysis of the DEGs of WT vs. Δ*SsZNC1* at 3 hpi and 9 hpi. (**A**) Gene ontology (GO) enrichment analysis of DEGs at 3 hpi. (**B**) GO enrichment analysis of DEGs at 9 hpi. (**C**) Kyoto Encyclopedia of Genes and Genomes (KEGG) enrichment analysis for DEGs at 3 hpi. (**D**) KEGG enrichment analysis for DEGs at 9 hpi.

To explore the potential genes regulated by SsZNC1 in *S. sclerotiorum*, an RNA-seq analysis was performed using three biological replicates of WT and *SsZNC1* knockout mutant (Δ*SsZNC1*-1) inoculated on *Arabidopsis* leaves at 3 hpi and 9 hpi, respectively. A total of 1187 differentially expressed genes (DEGs) were detected between the WT and Δ*SsZNC1*-1 mutant at 3 hpi and 9 hpi, of which 173 genes overlapped (Figure 4A). There were 387 DEGs between the WT and Δ*SsZNC1*-1 mutant at 3 hpi, including 158 up-regulated and 229 down-regulated genes (Figure 4B,C). The number of DEGs increased to 973 at 9 hpi, of which 427 were up-regulated and 546 were down-regulated (Figure 4B,D). These DEGs were used to classify the genes into a specific gene ontology (GO). The enriched GO terms at 3 hpi were predominantly associated with the glutathione metabolic process, extracellular region, monooxygenase activity, cellulose binding, and heme binding (Figure 5A). The enriched GO terms of DEGs at 9 hpi were mainly related to nucleolus, heme binding, RNA binding, cellular detoxification, and phosphopantetheine binding (Figure 5B). Five major identical GO terms were identified at both 3 hpi and 9 hpi, including the glutathione metabolic process, heme binding, cellular detoxification, phosphopantetheine binding, and methyltransferase activity (Figure 5A,B). Functional enrichment based on the KEGG pathways for the DEGs showed that they were related to multiple metabolic pathways, which included ribosome biogenesis in eukaryotes, valine, leucine, and isoleucine biosynthesis, tryptophan metabolism, starch and sucrose metabolism, glutathione metabolism, and so on (Figure 5C,D). These findings suggest dynamic molecular responses during the early stages of infection, implicating processes such as protein synthesis, energy metabolism, and cellular detoxification in the observed temporal changes. These results indicate that SsZNC1 plays important roles in multiple aspects of life activities during *S. sclerotiorum* infection.

### 3.6. SsZNC1 Positively Regulates the Expression of Virulence-Related Genes

**Figure 6:**
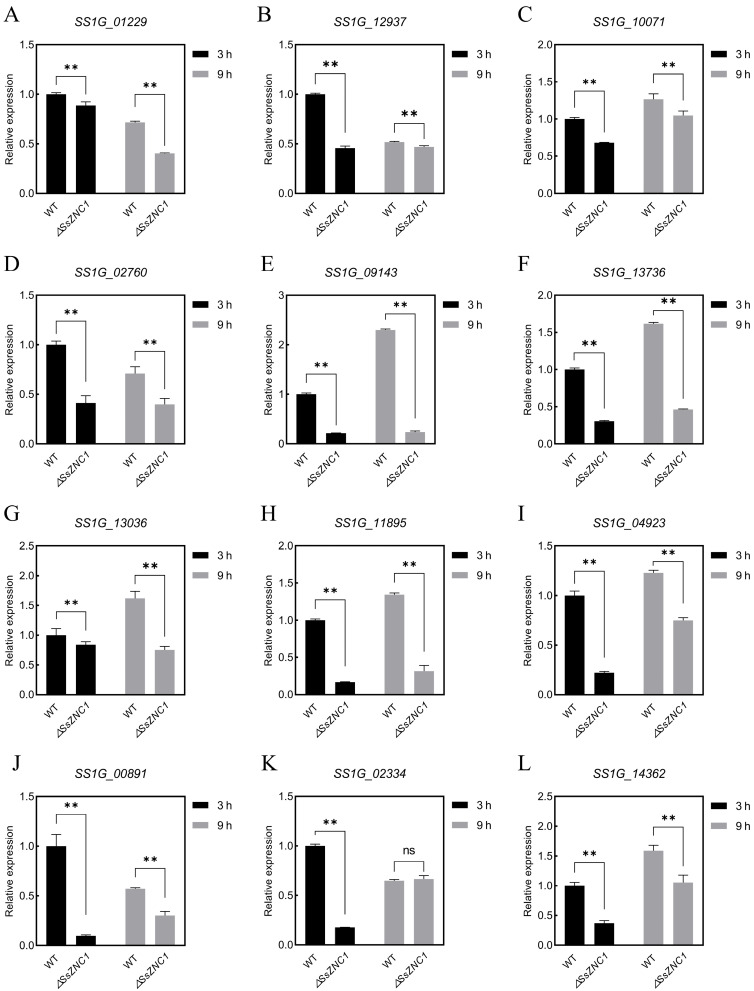
SsZNC1 influences the expression of secreted proteins and metabolism-related genes (**A**–**I**) RT-qPCR analysis of secreted proteins in WT and Δ*SsZNC1* at 3 hpi and 9 hpi. (**J**,**K**) RT-qPCR analysis of cellulose-catabolic-process-related genes. (**L**) RT-qPCR analysis of methyltransferase-activity-related genes. The constitutively expressed *β-tubulin* gene was used as the reference gene (**, *p* < 0.01; ns, no significance).

The transcriptomic analysis results revealed a significant down-regulation of the secreted proteins that may be involved in pathogenesis, including glycoside hydrolase family proteins, cutinase, pectinase, laccase, and hypothetical proteins, etc. The top nine DEGs with signal peptides were selected for an RT-qPCR analysis to validate the expression patterns identified in the transcriptome results. *SS1G_01229*, *SS1G_12937*, *SS1G_10071*, *SS1G_02760*, *SS1G_09143*, *SS1G_13736*, *SS1G_13036*, and *SS1G_11895* were predicted to be homologous to glycoside hydrolase, glycosyl hydrolase, pectin lyase, carbohydrate esterase, amidohydrolase, stress response protein, Cu-oxidase, and hydrophobin, respectively (Table S2). The expression pattern of each gene was significantly down-regulated at 3 hpi and 9 hpi (Figure 6A–I). Furthermore, we detected that *SS1G_00891*, *SS1G_02334*, and *SS1G_14362*, which are related to the cellulose catabolic process and methyltransferase activity, were significantly enriched in the transcriptome results, and their expression levels were also significantly down-regulated at 3 hpi and 9 hpi (Figure 6J–L). In addition, the expressions of some effectors known to be involved in pathogenesis and autophagy-related marker genes were also significantly decreased (Figure S3A–J). However, consistent with the transcriptome results, the expression patterns of genes in the early secretory pathway did not change significantly (Figure S3K,L). These results indicate that SsZNC1 affected the virulence of *S. sclerotiorum* by regulating the expressions of some virulence-related genes.

## 4. Discussion

Zn_2_Cys_6_ TFs are a large family characterized by the Zn_2_Cys_6_ binuclear cluster domain among pathogenic fungi, especially prevalent among ascomycetes [18,38]. Zn_2_Cys_6_ TFs have been characterized in diverse filamentous fungi [18,39,40,41]. However, there are relatively few studies on *S. sclerotiorum*, and there are still many genes in this family that have not been studied. Here, we identified an *S. sclerotiorum* Zn_2_Cys_6_ TF SsZNC1, which was significantly up-regulated during the infection period, based on public transcriptome data [29]. Consistently, we examined the expression pattern of *SsZNC1*, which was significantly elevated at 3 h post-inoculation (hpi) and gradually decreased after 9 hpi (Figure 1D), indicating that *SsZNC1* is involved in regulating the early stages of infection. Sequence alignment showed homologous of SsZNC1 in ascomycete fungi, suggesting its conservation and functional importance among various fungi (Figure 1A,B). By conducting a conserved motifs analysis of 250 homologous sequences, we found that there were three conserved motifs in *SsZNC1* (Figure 1C), suggesting that they may play important roles in the function of SsZNC1, especially the peptide TCKXRRIKCDE in motif 1. The protein size in the complemented strain was twice that expected (Figure S1D), and we hypothesized that homodimers were formed. This aligned with the result that Zn_2_Cys_6_ TFs generally bind in the form of a homodimer or heterodimer [42,43,44].

Unraveling the Zn_2_Cys_6_ TFs related to fungal virulence is crucial to understanding the pathogenesis of plant pathogens. However, there are relatively few studies on the Zn_2_Cys_6_ TF regulating *S. sclerotiorum* virulence. In our study, *SsZNC1* knockout mutants showed a reduced lesion area and relative biomass on *Arabidopsis* leaves and oilseed rape leaves (Figure 3), highlighting that SsZNC1 was important in regulating the virulence of *S. sclerotiorum*. To reveal important insights into the regulatory roles of SsZNC1, an RNA-seq analysis of the WT vs. Δ*SsZNC1* mutant was performed during an *S. sclerotiorum* infection of *Arabidopsis*. A total of 1187 DEGs were identified at 3 hpi and 9 hpi, of which 173 genes overlapped (Figure 4A), indicating consistent regulation by SsZNC1 across different time points. The DEGs within GO-encompassed processes included the glutathione metabolic process, heme binding, cellular detoxification, phosphopantetheine binding, and methyltransferase activity (Figure 5A,B). Methyltransferase activity and cellular detoxification were relevant to the virulence of plant pathogens [45,46,47,48]. The glutathione metabolic process, heme binding, and phosphopantetheine binding are involved in the biosynthesis and metabolism of fungi [49]. Significant KEGG pathway enrichments, encompassing ribosome biogenesis, valine, leucine, and isoleucine biosynthesis, pentose and glucuronate interconversions, tryptophan metabolism, starch and sucrose metabolism, glutathione metabolism, and amino sugar and nucleotide sugar metabolism, underscore the multifaceted impact of SsZNC1 on critical cellular functions (Figure 5C,D). In addition, the down-regulation of virulence-related secreted proteins, effectors, and autophagy-related genes from the transcriptome data was verified through RT-qPCR analysis (Figure 6 and Figure S3). The secreted proteins analyzed by RT-qPCR included glycoside hydrolase family proteins, cutinase, pectinase, and laccase, and are thought to be related to the virulence of the pathogens [50,51,52,53]. The autophagy-related genes *SsATG1*, *SsATG8*, and *SsATG13* were also required for virulence in *S. sclerotiorum* [54,55,56]. These results indicate that SsZNC1 regulated the virulence of *S. sclerotiorum* through affecting the expressions of genes related to metabolic pathways, biosynthetic pathways, secreted proteins, and autophagy. Consistently, previous studies have shown that Zn_2_Cys_6_ TFs were involved in the virulence by regulating diverse pathways. Pf2, an extensively researched Zn_2_Cys_6_ TF, was a key regulator for controlling the necrotrophic lifestyle and virulence in many pathogenic fungi [57,58]. Pf2 played a regulatory role similar to SsZNC1, influencing pathogen virulence by modulating the expression of putative-effector-like proteins and plant-cell wall-degrading enzymes during early infection [59]. *Colletotrichum orbiculare* Zn_2_Cys_6_ TF Mtf4 regulated appressorium development and reduced virulence through the MOR kinase signaling pathway in response to host-derived cutin monomers [60]. These studies demonstrated that Zn_2_Cys_6_ TFs affected the virulence of pathogens by regulating diverse pathways.

Increasing evidence has indicated that TFs play important roles in the growth, development, and response to abiotic stress of necrotrophic phytopathogens [16,24,25,61]. In our study, the targeted deletion of *SsZNC1* resulted in mutants with altered sclerotial development (Figure 2C–E) and weak growth on PDA containing high concentrations of sorbitol (Figure 2F,G), demonstrating the importance of SsZNC1 in regulating sclerotial development and hyperosmotic stress response. These findings provide insights into the regulatory network orchestrated by SsZNC1, shedding light on its pivotal roles in the life activities of *S. sclerotiorum*. While a significant number of DEGs were discovered in our analysis, the specific genes directly regulated by SsZNC1 remain unknown. Further investigation is required to unravel the direct targets of SsZNC1 and elucidate the molecular mechanisms underlying its regulatory functions.

In summary, SsZNC1, as a Zn_2_Cys_6_ TF of *S. sclerotiorum*, played an important role in regulating the virulence, sclerotial development, and tolerance of hyperosmotic stress in *S. sclerotiorum*. We preliminarily explained the pathways regulated by SsZNC1 through a transcriptomic analysis. Our results are helpful for understanding the role of SsZNC1 in the virulence, development, and response to environmental stresses of *S. sclerotiorum*, and helping to find its potential direct regulatory pathways.

## 5. Conclusions

In this study, we identified and characterized a Zn_2_Cys_6_ TF, SsZNC1, in *S. sclerotiorum*, and found that SsZNC1 is a positive regulator of virulence, sclerotial development, and osmotic stress response. Based on the transcriptomic analysis results, it is evident that SsZNC1 plays a crucial role in shaping multiple key cellular functions, including the cellulose catabolic process, methyltransferase activity, and pathogenicity, etc. Our results revealed a potential mechanism by which SsZNC1 regulated virulence, development, and response to abiotic stresses in *S. sclerotiorum*.

## Data Availability

Data are contained within the article and Supplementary Materials.

## Supplementary Materials

The following supporting information can be downloaded at: https://www.mdpi.com/article/10.3390/jof10020135/s1, Figure S1: Analysis of *SsZNC1* transformants; Figure S2: Survivability of the WT and *SsZNC1* transformants in stress media; Figure S3: Validation of the RNA-seq data by RT-qPCR; Table S1: Primers used in this study; Table S2: The information of genes used for RNA-Seq analysis.

## References

[1] Boland G., Hall R. (1994). Index of plant hosts of *Sclerotinia sclerotiorum*. Can. J. Plant Pathol..

[2] Liang X., Rollins J.A. (2018). Mechanisms of broad host range necrotrophic pathogenesis in *Sclerotinia sclerotiorum*. Phytopathology.

[3] Bolton M.D., Thomma B.P., Nelson B.D. (2006). *Sclerotinia sclerotiorum* (Lib.) de Bary: Biology and molecular traits of a cosmopolitan pathogen. Mol. Plant Pathol..

[4] Suszkiw J. (2007). ARS-led effort tackles white mold. Agric. Res..

[5] Xu L., Li G., Jiang D., Chen W. (2018). *Sclerotinia sclerotiorum*: An evaluation of virulence theories. Annu. Rev. Phytopathol..

[6] Merriman P. (1976). Survival of sclerotia of *Sclerotinia sclerotiorum* in soil. Soil Biol. Biochem..

[7] Hossain M.M., Sultana F., Li W., Tran L.S.P., Mostofa M.G. (2023). *Sclerotinia sclerotiorum* (Lib.) de Bary: Insights into the pathogenomic features of a global pathogen. Cells.

[8] Derbyshire M.C., Newman T.E., Khentry Y., Owolabi Taiwo A. (2022). The evolutionary and molecular features of the broad-host-range plant pathogen *Sclerotinia sclerotiorum*. Mol. Plant Pathol..

[9] Yang Y., Steidele C.E., Rössner C., Löffelhardt B., Kolb D., Leisen T., Zhang W., Ludwig C., Felix G., Seidl M.F. (2023). Convergent evolution of plant pattern recognition receptors sensing cysteine-rich patterns from three microbial kingdoms. Nat. Commun..

[10] Xie C., Shang Q., Mo C., Xiao Y., Wang G., Xie J., Jiang D., Xiao X. (2021). Early secretory pathway-associated proteins SsEmp24 and SsErv25 are involved in morphogenesis and pathogenicity in a filamentous phytopathogenic fungus. mBio.

[11] Isbel L., Grand R.S., Schübeler D. (2022). Generating specificity in genome regulation through transcription factor sensitivity to chromatin. Nat. Rev. Genet..

[12] Shelest E. (2008). Transcription factors in fungi. FEMS Microbiol. Lett..

[13] Guo X., Atehli D., Chen M., Chen D., Wang Y. (2023). A Zn(II)(2)Cys(6) transcription factor MPsGeI suppresses pigment biosynthesis in *Monascus*. Int. J. Biol. Macromol..

[14] John E., Singh K.B., Oliver R.P., Tan K.C. (2021). Transcription factor control of virulence in phytopathogenic fungi. Mol. Plant Pathol..

[15] Urnov F.D. (2002). A feel for the template: Zinc finger protein transcription factors and chromatin. Biochem. Cell Biol..

[16] Lu S., Zhang X., He C., Li G., Chen T., Li B., Tian S., Zhang Z. (2023). The Zn (II) _2_Cys_6_ transcription factor BcDIC affects the asexual reproduction of *Botrytis cinerea* by regulating pectinesterase genes. Phytopathol. Res..

[17] Zhang W.Q., Gui Y.J., Short D.P., Li T.G., Zhang D.D., Zhou L., Liu C., Bao Y.M., Subbarao K.V., Chen J.Y. (2018). *Verticillium dahliae* transcription factor VdFTF1 regulates the expression of multiple secreted virulence factors and is required for full virulence in cotton. Mol. Plant Pathol..

[18] Liu X., Li R., Zeng Q., Li Y., Chen X. (2023). A novel Zn_2_Cys_6_ transcription factor, TopC, positively regulates trichodin A and asperpyridone A biosynthesis in *Tolypocladium ophioglossoides*. Microorganisms.

[19] Delorme-Axford E., Wen X., Klionsky D.J. (2023). The yeast transcription factor Stb5 acts as a negative regulator of autophagy by modulating cellular metabolism. Autophagy.

[20] Li J., Mu W., Veluchamy S., Liu Y., Zhang Y., Pan H., Rollins J.A. (2018). The GATA-type IVb zinc-finger transcription factor SsNsd1 regulates asexual–sexual development and appressoria formation in *Sclerotinia sclerotiorum*. Mol. Plant Pathol..

[21] Li J., Zhang X., Li L., Liu J., Zhang Y., Pan H. (2018). Proteomics analysis of SsNsd1-mediated compound appressoria formation in *Sclerotinia sclerotiorum*. Int. J. Mol. Sci..

[22] Zhu G., Yu G., Zhang X., Liu J., Zhang Y., Rollins J.A., Li J., Pan H. (2019). The formaldehyde dehydrogenase SsFdh1 is regulated by and functionally cooperates with the GATA transcription factor SsNsd1 in *Sclerotinia sclerotiorum*. MSystems.

[23] Jiao W., Yu H., Cong J., Xiao K., Zhang X., Liu J., Zhang Y., Pan H. (2022). Transcription factor SsFoxE3 activating *SsAtg8* is critical for sclerotia, compound appressoria formation, and pathogenicity in *Sclerotinia sclerotiorum*. Mol. Plant Pathol..

[24] Fan H., Yu G., Liu Y., Zhang X., Liu J., Zhang Y., Rollins J.A., Sun F., Pan H. (2017). An atypical forkhead-containing transcription factor SsFKH1 is involved in sclerotial formation and is essential for pathogenicity in *Sclerotinia sclerotiorum*. Mol. Plant Pathol..

[25] Cong J., Xiao K., Jiao W., Zhang C., Zhang X., Liu J., Zhang Y., Pan H. (2022). The coupling between cell wall integrity mediated by MAPK kinases and SsFkh1 is involved in sclerotia formation and pathogenicity of *Sclerotinia sclerotiorum*. Front. Microbiol..

[26] Xu T., Li J., Yu B., Liu L., Zhang X., Liu J., Pan H., Zhang Y. (2018). Transcription factor SsSte12 was involved in mycelium growth and development in *Sclerotinia sclerotiorum*. Front. Microbiol..

[27] Qu X., Yu B., Liu J., Zhang X., Li G., Zhang D., Li L., Wang X., Wang L., Chen J. (2014). MADS-box transcription factor SsMADS is involved in regulating growth and virulence in *Sclerotinia sclerotiorum*. Int. J. Mol. Sci..

[28] Liu L., Lyu X., Pan Z., Wang Q., Mu W., Benny U., Rollins J.A., Pan H. (2022). The C2H2 transcription factor SsZFH1 regulates the size, number, and development of apothecia in *Sclerotinia sclerotiorum*. Phytopathology.

[29] Peyraud R., Mbengue M., Barbacci A., Raffaele S. (2019). Intercellular cooperation in a fungal plant pathogen facilitates host colonization. Proc. Natl. Acad. Sci. USA.

[30] Wang J., Chitsaz F., Derbyshire M.K., Gonzales N.R., Gwadz M., Lu S., Marchler G.H., Song J.S., Thanki N., Yamashita R.A. (2023). The conserved domain database in 2023. Nucleic Acids Res..

[31] Knudsen S. (1999). Promoter2. 0: For the recognition of PolII promoter sequences. Bioinformatics.

[32] Tamura K., Stecher G., Kumar S. (2021). MEGA11: Molecular evolutionary genetics analysis version 11. Mol. Biol. Evol..

[33] Rivero L., Scholl R., Holomuzki N., Crist D., Grotewold E., Brkljacic J. (2014). Handling *Arabidopsis* plants: Growth, preservation of seeds, transformation, and genetic crosses. Arab. Protoc..

[34] Catlett N.L., Lee B.N., Yoder O., Turgeon B.G. (2003). Split-marker recombination for efficient targeted deletion of fungal genes. Fungal Genet. Rep..

[35] Rollins J.A. (2003). The *Sclerotinia sclerotiorum pac1* gene is required for sclerotial development and virulence. Mol. Plant-Microbe Interact..

[36] Aleksander S.A., Balhoff J., Carbon S., Cherry J.M., Drabkin H.J., Ebert D., Feuermann M., Gaudet P., Harris N.L. (2023). The Gene Ontology knowledgebase in 2023. Genetics.

[37] Kanehisa M., Furumichi M., Sato Y., Kawashima M., Ishiguro-Watanabe M. (2023). KEGG for taxonomy-based analysis of pathways and genomes. Nucleic Acids Res..

[38] Todd R.B., Zhou M., Ohm R.A., Leeggangers H.A., Visser L., De Vries R.P. (2014). Prevalence of transcription factors in ascomycete and basidiomycete fungi. BMC Genom..

[39] Chen Y., Yuan G., Hsieh S., Lin Y., Wang W., Liaw L., Tseng C. (2010). Identification of the *mokH* gene encoding transcription factor for the upregulation of monacolin K biosynthesis in *Monascus pilosus*. J. Agric. Food Chem..

[40] Bergmann S., Schümann J., Scherlach K., Lange C., Brakhage A.A., Hertweck C. (2007). Genomics-driven discovery of PKS-NRPS hybrid metabolites from *Aspergillus nidulans*. Nat. Chem. Biol..

[41] Abe Y., Ono C., Hosobuchi M., Yoshikawa H. (2002). Functional analysis of *mlcR*, a regulatory gene for ML-236B (compactin) biosynthesis in *Penicillium citrinum*. Mol. Genet. Genom..

[42] Joshua I.M., Höfken T. (2017). From lipid homeostasis to differentiation: Old and new functions of the zinc cluster proteins Ecm22, Upc2, Sut1 and Sut2. Int. J. Mol. Sci..

[43] Yang H., Tong J., Lee C.W., Ha S., Eom S.H., Im Y.J. (2015). Structural mechanism of ergosterol regulation by fungal sterol transcription factor Upc2. Nat. Commun..

[44] Schjerling P., Holmberg S. (1996). Comparative amino acid sequence analysis of the C_6_ zinc cluster family of transcriptional regulators. Nucleic Acids Res..

[45] Molina L., Kahmann R. (2007). An *Ustilago maydis* gene involved in H_2_O_2_ detoxification is required for virulence. Plant Cell.

[46] Ding C., Festa R.A., Chen Y.L., Espart A., Palacios Ò., Espín J., Capdevila M., Atrian S., Heitman J., Thiele D.J. (2013). *Cryptococcus neoformans* copper detoxification machinery is critical for fungal virulence. Cell Host Microbe.

[47] Bayram O., Krappmann S., Ni M., Bok J.W., Helmstaedt K., Valerius O., Braus-Stromeyer S., Kwon N.-J., Keller N.P., Yu J.-H. (2008). VelB/VeA/LaeA complex coordinates light signal with fungal development and secondary metabolism. Science.

[48] Ahmed Y.L., Gerke J., Park H.S., Bayram Ö., Neumann P., Ni M., Dickmanns A., Kim S.C., Yu J.H., Braus G.H. (2013). The velvet family of fungal regulators contains a DNA-binding domain structurally similar to NF-κB. PLoS Biol..

[49] Thomas P.D., Ebert D., Muruganujan A., Mushayahama T., Albou L.P., Mi H. (2022). PANTHER: Making genome-scale phylogenetics accessible to all. Protein Sci..

[50] Reignault P., Valette-Collet O., Boccara M. (2008). The importance of fungal pectinolytic enzymes in plant invasion, host adaptability and symptom type. Eur. J. Plant Pathol..

[51] Feng B., Li P., Fu L., Yu X. (2015). Exploring laccase genes from plant pathogen genomes: A bioinformatic approach. Genet. Mol. Res.

[52] Rafiei V., Vélëz H., Tzelepis G. (2021). The role of glycoside hydrolases in phytopathogenic fungi and oomycetes virulence. Int. J. Mol. Sci..

[53] Gong Y., Fu Y., Xie J., Li B., Chen T., Lin Y., Chen W., Jiang D., Cheng J. (2022). *Sclerotinia sclerotiorum* SsCut1 modulates virulence and cutinase activity. J. Fungi.

[54] Kabbage M., Williams B., Dickman M.B. (2013). Cell death control: The interplay of apoptosis and autophagy in the pathogenicity of *Sclerotinia sclerotiorum*. PLoS Path..

[55] Zhang H., Li Y., Lai W., Huang K., Li Y., Wang Z., Chen X., Wang A. (2021). SsATG8 and SsNBR1 mediated-autophagy is required for fungal development, proteasomal stress response and virulence in *Sclerotinia sclerotiorum*. Fungal Genet. Biol..

[56] Jiao W., Yu H., Chen X., Xiao K., Jia D., Wang F., Zhang Y., Pan H. (2022). The *SsAtg1* activating autophagy is required for sclerotia formation and pathogenicity in *Sclerotinia sclerotiorum*. J. Fungi.

[57] Cho Y., Ohm R.A., Grigoriev I.V., Srivastava A. (2013). Fungal-specific transcription factor AbPf2 activates pathogenicity in *Alternaria brassicicola*. Plant J..

[58] Rybak K., See P.T., Phan H.T., Syme R.A., Moffat C.S., Oliver R.P., Tan K.C. (2017). A functionally conserved Zn_2_Cys_6_ binuclear cluster transcription factor class regulates necrotrophic effector gene expression and host-specific virulence of two major Pleosporales fungal pathogens of wheat. Mol. Plant Pathol..

[59] Jones D.A., John E., Rybak K., Phan H.T., Singh K.B., Lin S.Y., Solomon P.S., Oliver R.P., Tan K.-C. (2019). A specific fungal transcription factor controls effector gene expression and orchestrates the establishment of the necrotrophic pathogen lifestyle on wheat. Sci. Rep..

[60] Kodama S., Nishiuchi T., Kubo Y. (2019). *Colletotrichum orbiculare* MTF4 is a key transcription factor downstream of MOR essential for plant signal-dependent appressorium development and pathogenesis. Mol. Plant-Microbe Interact..

[61] Schumacher J., Simon A., Cohrs K.C., Viaud M., Tudzynski P. (2014). The transcription factor BcLTF1 regulates virulence and light responses in the necrotrophic plant pathogen *Botrytis cinerea*. PLoS Genet..

