# Supplementary material for: The Transcription Factor SsZNC1 Mediates Virulence, Sclerotial Development, and Osmotic Stress Response in Sclerotinia sclerotiorum
Source: J Fungi (Basel). 2024 Feb 8;10(2):135. doi: 10.3390/jof10020135 (PMC10890190; doi:10.3390/jof10020135)
Supplement: Supplementary file 1 [file jof-10-00135-s001.zip › jof-2841628-supplementary.pdf]

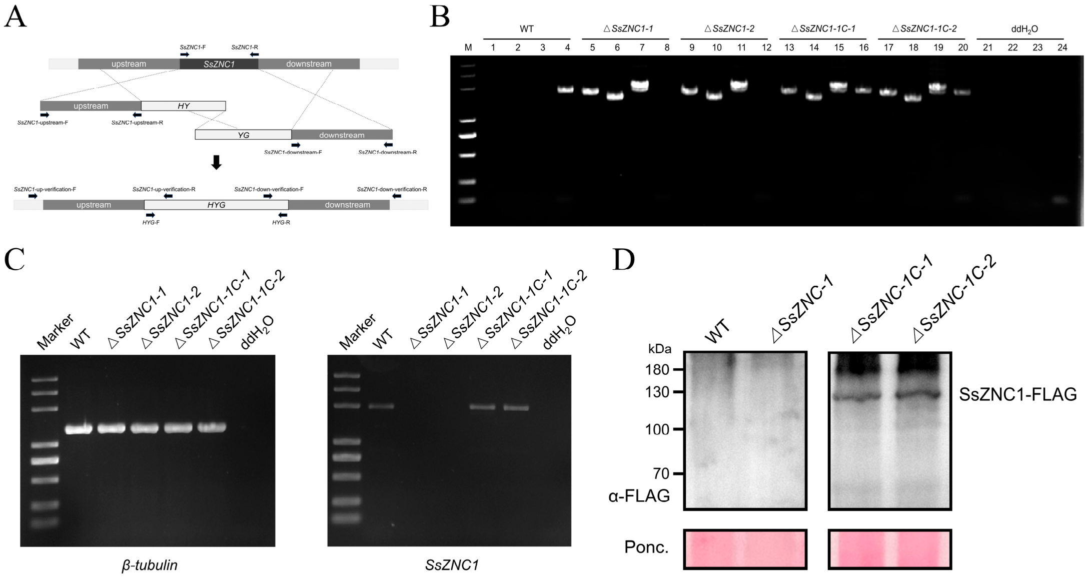

Figure S1. Analysis of *SsZNC1* transformants. **(A)** Schematic diagram of the *SsZNC1* gene knock-out strategy. **(B)** PCR validation of the *SsZNC1* knockout mutants and complemented strain. The verification of *SsZNC1* upstream (Lanes 1, 5, 9, 13, 17, 21); the verification of *SsZNC1* downstream (Lanes 2, 6, 10, 14, 18, and 22); the verification of *HYG* (Lanes 3, 7, 11, 15, 19, and 23); the verification of the full-length *SsZNC1* gene (Lanes 4, 8, 12, 16, 20, and 24). Lane M, DL5000 marker (3428Q, Takara, China). **(C)** RT-PCR analysis of the transcription of *SsZNC1* in different strains. The *β-tubulin* gene in *S. sclerotiorum* was used as the reference gene. Lane Marker, DL5000 marker. Primer sequences used were listed in Table S1. **(D)** Detection of protein of *SsZNC1* transformants by western blot. The protein expression of transformants through western blot using FLAG antibody (66008-4-Ig, Proteintech, China).

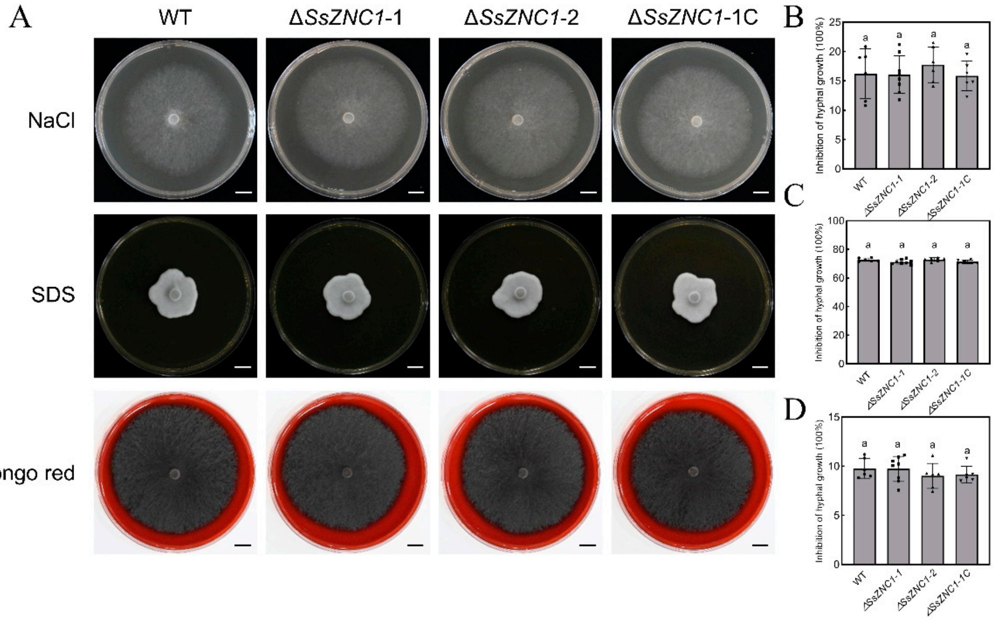

Figure S2. Survivability of the WT and *SsZNC1* transformants in stress media. **(A)** Growth observation of the WT and the *SsZNC1* transformants on PDA medium containing 0.5 M NaCl, 0.02% SDS, and 2.5 mg/mL Congo red. Bar, 1 cm. **(B-D)** The inhibition of hyphal growth of the WT and the *SsZNC1* transformants on PDA medium containing different stress factors. The values represent means derived from three independent replicates, and the error bars indicate ± SD of the mean.

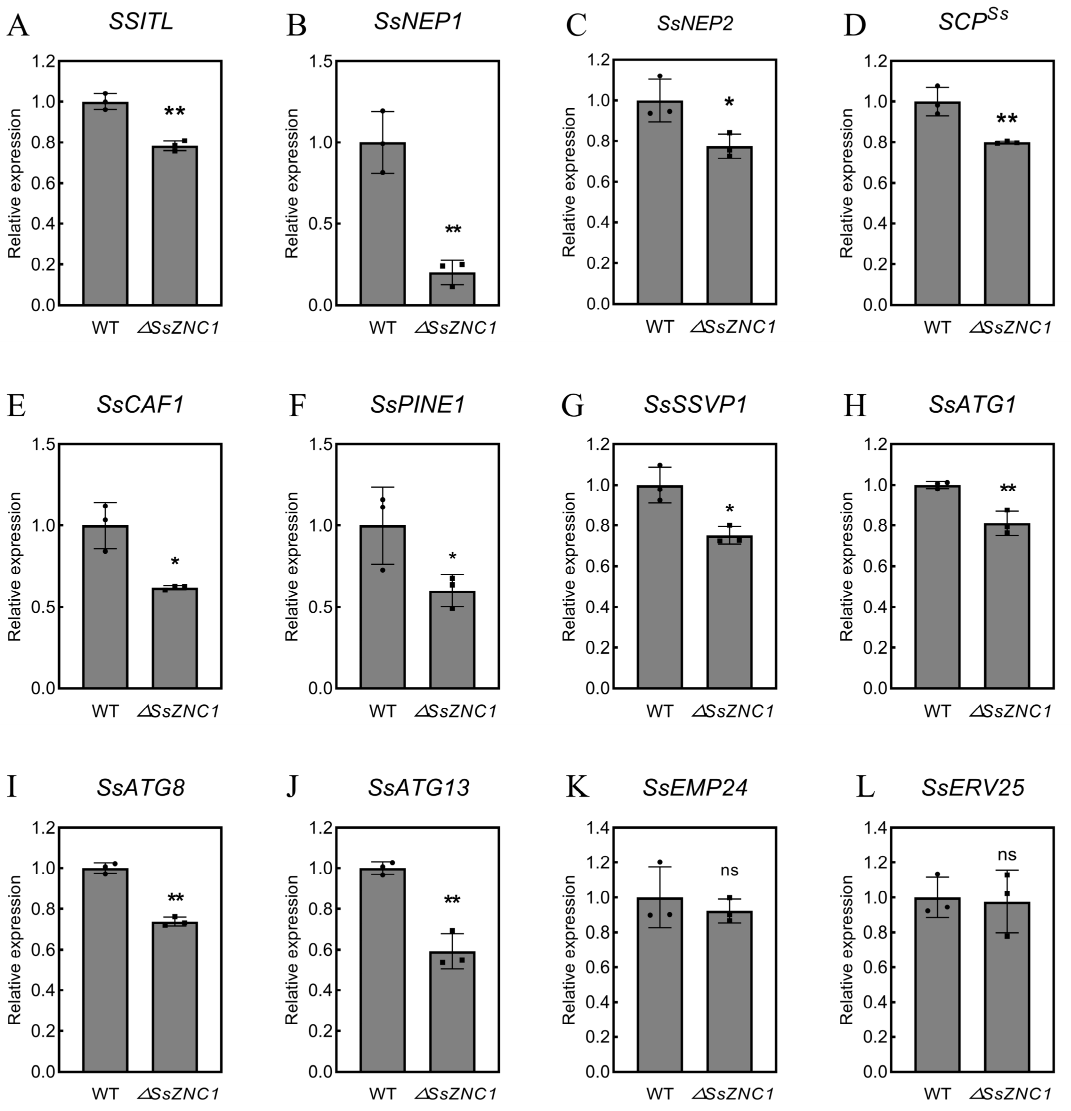

Figure S3. Validation of the RNA-seq data by RT-qPCR. **(A-G)** The relative expression levels of effectors were verified by RT-qPCR at 3 hpi. **(H-J)** The relative expression levels of autophagy related genes were verified by RT-qPCR at 3 hpi. **(K, L)** The relative expression levels of early secretory pathway related genes were verified by RT-qPCR at 3 hpi. The constitutively expressed *β-tubulin* gene was used as the reference gene to standardize data (*, $P < 0.05$; **, $P < 0.01$; ns, no significance).

Table S1. Primers used in this study.

Name	Fwd Primer	Rvs Primer	Description of primers
SsZNC1	atgcttaacttgcaagtaagtcgatgc	tcaaggggatgacctaacaggc	Amplification of *SsZNC1* for sequencing
SsZNC1-promoter	tggaacaacaagtgaagaac	ctaatgagcgactgagaca	Amplification of *SsZNC1* promoter
HYG	cagaagatgatattgaaggagcac	aaagaaggattacctctaaacaagtgta	Amplification of hygromycin-resistant gene
SsZNC1-upstream	ccaaaccgaacgacaatccg	ccggatcgcaccttggg	Amplification of *SsZNC1* upstream
SsZNC1-downstream	gctcgagcttgagcttgattg	agtgagtgctgccagaagagg	Amplification of *SsZNC1* downstream
SsZNC1-up-verification	ataaacgacgctggtcacgc	cgccgccgctactgctacaagtg	Amplification of the upstream of *SsZNC1* overlapped PtrpC for verification of deletion mutant
SsZNC1-down-verification	ctggaaagcaacaccgtgaact	agactgaggaatccgctcttgg	Amplification of the downstream of *SsZNC1* overlapped TrpC for verification of deletion mutant
qPCR-*SsTubulin*	acctccatccaagaactc	gaactccatctcgtccat	*SS1G_04652* (*β-Tubulin*) for relative biomass
qPCR-*AtUBQ10*	agatccaggacaaggaaggtattc	cgcaggaccaagtgaagagtag	*AT4G05320* (*UBQ10*) for relative biomass
qPCR-*SsZNC1*	tggaacaacaagtgaagaac	ctaatgagcgactgagaca	*SsZNC1* expression assary
qPCR-*SS1G_09143*	cggattggtatcgtcagattgt	cgttaagaagtcgtgctaatgc	*SS1G_09143* expression assary
qPCR-*SS1G_04923*	gcggaagcgattattggtctg	ggttgagcaggcgaaggata	*SS1G_04923* expression assary
qPCR-*SS1G_01229*	tcggcactggagttgaacat	agcattggagcaggtggtag	*SS1G_01229* expression assary
qPCR-*SS1G_11895*	ctctcatctgccgctatggt	tgattcctccgctggtatctc	*SS1G_11895* expression assary
qPCR-*SS1G_10071*	cacatcggtcgtcaacacatt	caggtagcagagtaatcggaga	*SS1G_10071* expression assary
qPCR-*SS1G_12937*	gatggtggaatgctgcgaat	ccgtggcgttggtagaatc	*SS1G_12937* expression assary
qPCR-*SS1G_13036*	accttccacagccaactatga	ttactagcaccgtcttctccaa	*SS1G_13036* expression assary

Name	Fwd Primer	Rvs Primer	Description of primers
qPCR-*SS1G_02760*	cctccaccatctccaccatc	gccttcatagccgcaacac	*SS1G_02760* expression assary
qPCR-*SS1G_13736*	ttcatcggtcgcttcagttg	ctccagtagtcgtcggtgta	*SS1G_13736* expression assary
qPCR-*SS1G_00891*	ctcagttcaggcagtggttac	cggttagtatggcttgtcttcc	*SS1G_00891* expression assary
qPCR-*SS1G_02334*	ttgctgagatggatgctgatg	tgaccgttgatgaacttgatgt	*SS1G_02334* expression assary
qPCR-*SS1G_14362*	ggacacctgaagaataccaagt	ccatcggcttacatccatatac	*SS1G_14362* expression assary
qPCR-SCP^{Ss}	aatctcctacgccatccaa	cgcagcacaggtataagc	SCP^{Ss} expression assary
qPCR-*SsCAF1*	tggcttgtgtaggattgg	cgaagttgtctatgtgatgt	*SsCAF1* expression assary
qPCR-*SsITL*	ctcaggaagataacggaaca	catcaccatcatagtcatacac	*SsITL* expression assary
qPCR-*SsNEP1*	gtcaacctccataccaaca	acagttagcatcaccgaat	*SsNEP1* expression assary
qPCR-*SsNEP2*	cacactctatgaagcctaca	ccattgctactgccactt	*SsNEP2* expression assary
qPCR-*SsPINE1*	attctcgcagcatcaact	atccgtcaactaatccatca	*SsPINE1* expression assary
qPCR-*SsVP1*	atggcaatcctgtcaagtat	ggtcttcttatcgcacatatc	*SsVP1* expression assary
qPCR-*SsATG1*	tcatacactaccgccattag	taacctctccaccatcaca	*SsATG1* expression assary
qPCR-*SsATG13*	acactccagccatacctt	catcttctaaccttgcctct	*SsATG13* expression assary
qPCR-*SsATG8*	ggtcaattcgtctatgtcatc	ggcttcttctaaggcttca	*SsATG8* expression assary
qPCR-*SsEMP24*	ttcacggcattgtctatgt	cttcaccaaccagaactaac	*SsEMP24* expression assary
qPCR-*SsERV25*	cattgtaggcgagaagagaa	gttgtgaatacggcttgtg	*SsERV25* expression assary

Table S2. The information of genes used for RNA-Seq analysis.

**Gene ID**	**Locus tag**	**Chromosomal location**	**SignalP4**	**TM**	**Gene function description**
SS1G_01229	Sscle01g010410	Chromosome 1 (3541576..3544299)	YES	No TM	Glycoside hydrolase family 55 protein (FCW)
SS1G_12937	Sscle02g011730	Chromosome 2 (47473..48702)	YES	No TM	Glycosyl hydrolase 76 (mannans degradation)
SS1G_10071	Sscle16g107500	Chromosome 16 (46815..48014)	YES	No TM	Pectin lyase (pectin degradation)
SS1G_02760	Sscle04g036990	Chromosome 4 (1709458..1710439)	YES	TM	Carbohydrate esterase family 5 protein (hemicellulose degradation)
SS1G_09143	Sscle15g107260	Chromosome 15 (1740933..1742517)	YES	No TM	Amidohydrolase 2
SS1G_13736	Sscle10g079780	Chromosome 10 (1741791..1743368)	YES	No TM	Stress response protein rds1(ferritin 2)
SS1G_13036	Sscle02g021570	Chromosome 2 (3422968..3425247)	YES	No TM	Cu-oxidase 3
SS1G_11895	Sscle12g090620	Chromosome 12 (1405870..1406372)	YES	No TM	Hydrophobin class I
SS1G_04923	Sscle08g062810	Chromosome 8 (258861..260207)	YES	No TM	Hypothetical protein
SS1G_00891	Sscle03g023660	Chromosome 3 (604987..606652)	NO	No TM	Cellulose catabolic process and cellulose binding
SS1G_02334	Sscle04g033820	Chromosome 4 (604987..606652)	YES	No TM	Cellulose catabolic process and cellulose binding
SS1G_14362	Sscle16g110770	Chromosome 16(1227771..1230138)	NO	No TM	Methyltransferase activity
